# Emotional Peer Support Interventions for Students With SEND: A Systematic Review

**DOI:** 10.3389/fpsyg.2021.797913

**Published:** 2021-12-28

**Authors:** Kevin van der Meulen, Laura Granizo, Cristina del Barrio

**Affiliations:** ^1^Departamento de Psicología Evolutiva y de la Educación, Facultad de Psicología, Universidad Autónoma de Madrid, Madrid, Spain; ^2^Facultad de Ciencias de la Salud y la Educación, Universidad a Distancia de Madrid, Madrid, Spain

**Keywords:** peer support, SEND, school ethos, student participation, peer interaction, emotional support

## Abstract

Emotional peer support systems have benefits for student-student relationships and allow for children and adolescents' participation in schools. For students with specific educational needs and disabilities (SEND), positive relationships seem to be more difficult to attain and these students are more vulnerable to suffer negative peer experiences such as bullying and social exclusion. Systems in which peers can show helpful behavior are beneficial for schools in order to create a positive, supportive climate. Emotional peer support entails social interaction through emotional or practical help based on what these peers have in common and many times with benefits for both. This systematic review identified interventions of emotional peer support in schools for students with SEND. Twenty-three studies were identified that involved four types of befriending: circle of friends, peer buddying, peer networks, and social lunch clubs. Studies reported mainly positive outcomes for both focus students and peer supporters in terms of increased social interaction and social acceptance, as well as enhanced self-esteem and empathy on the individual level. Further bonding of the students by friendship was also perceived, but more precise data is required to draw further conclusions. Support by the school as an institution, the specific role of the teacher, and family participation are important factors related to the impact of peer support systems. Information on these aspects was scarce, and it is recommended to include variables of this nature in future research. Intervention descriptions revealed students' active participation through suggestions for activities, however their involvement in organizing the systems was limited. More research is needed to learn about the opportunities of emotional peer support to improve student-student relationships including the active involvement of the peers themselves in this support.

## Introduction

Peer interaction and the building of positive peer relationships are essential for young people's development (Piaget, 1932; Hartup, 1979, 1996; Johnson, 1980). However, for students with specific educational needs and disabilities (SEND), positive relationships seem to be more difficult to attain. They are more vulnerable to being bullied or suffer from social exclusion in school (Thompson et al., 1994; Carter and Spencer, 2006) including after transitioning from primary to secondary school (Hughes et al., 2013). Students with autism spectrum conditions (ASC) seem to be more at risk for being bullied than students with other types of SEND (Humphrey and Hebron, 2015). Moreover, Lasgaard et al. (2010) found that adolescents with ASC reported often or always having feelings of loneliness. Feeling lonely was associated with a lack of social support from classmates. Therefore, the need to improve peer experiences in schools for students with SEND has been underlined by many researchers and practitioners.

Over the last decades, scholars have highlighted the importance of the involvement of the peer group in promoting positive student-student relationships (e.g., Johnson et al., 2010), and reducing bullying and social exclusion in schools (Salmivalli, 1999). Peers show qualities that make them more effective agents than adults in schools: they share the same status, and are therefore more easily accessible and more influential, among other reasons.

In literature on school ethos there are references to students taking an active role in decision-making or norms settlement in their schools, as well as to the benefits of these practices. Some of these experiences are based on the Just Community Schools approach (Power et al., 1989). Another example is The Three R action (*rights, respect, responsibility*). On the basis of The Three R action, Covell et al. (2008) conclude that the combination of actions promoting participation together with students' knowledge about their rights help them act as mature citizens. Students' participation in areas affecting them directly is one of the rights of children (United Nations, 1989), has positive consequences for them along with the school community (Edwards and Mullis, 2003; Saiz-Linares et al., 2019), and prepares them for social commitment (Flanagan et al., 1998; Haste, 2005), especially when pupils' actions respond to social environmental needs, as in service-learning experiences (Hart et al., 2006; Traver-Martí et al., 2019). However, adults in schools seem to not be prone to sharing power with pupils. Students themselves report on their low participation level in school (Coiduras et al., 2016) and point to a more reactive nature by merely deciding on ideas and proposals that come from teachers and which are rather non-essential matters. Many times decisions are taken hierarchically, and sometimes students feel pressure to accept these in a non-voluntary way (Granizo et al., 2019).

The creation of systems in which peers can show helpful behavior is beneficial for schools in order to build a positive, supportive climate. Peer support is about social interactions that involve giving information, emotional or practical help based on what peers have in common and many times with benefits for both (Cowie and Wallace, 2000). Various types of peer support systems exist, which can be broadly divided into two categories (Cowie and Wallace, 2000). The first involves methods related to education and information-giving, such as peer tutoring and mentoring. The contents of the support are mainly academic such as reading activities or mathematical problems. There is a considerable amount of literature available on this type of peer support, including analyses on the effectiveness of their application with SEND students, for example peer tutoring (Talbott et al., 2017; Alzahrani and Leko, 2018).

The second category involves emotional support given by peers to others who are in need, and refers to befriending, mediation/conflict resolution, and counseling-based approaches (Cowie and Wallace, 2000). Befriending implies support in various formats, such as offering companionship to students perceived as solitary or helping peers who are bullied or find it hard to make friends. Conflict resolution entails mediating between peers, or a peer and an adult, who are in disagreement. Counseling-based interventions require more extended training in counseling skills. Students ask for help to one of the counselors directly or the student contacts the service and is referred to a counselor. All these types of emotional peer support systems share several aspects (Sharp and Cowie, 1998). For a start, they require a recruitment or selection process. Students receive training focused on listening and communication skills, empathy for peers with social or emotional difficulties and problem-solving strategies (Cowie and Sharp, 1996; Cowie and Wallace, 2000). Adults are in charge of this training and maintain a supervisory but non-directive role during the intervention. The peers themselves fulfill the most important role and have the capacity to manage the helping practices. And finally, as it typically takes place outside the classroom, whether the support system succeeds or fails depends on the commitment of the students who volunteer for it.

The effectiveness of emotional peer support has been demonstrated in several general population studies in secondary schools, with respect to counseling-based systems (Naylor and Cowie, 1999; Cowie et al., 2002; Houlston and Smith, 2009; Del Barrio et al., 2011). Results showed these were efficient in terms of the helping process; students who were supported by peers reported feeling emotional relief and an increased ability to cope with problems. Benefits for peer supporters included increased self-esteem and communication skills.

Peer support in schools is more effective when it is integrated into a whole school supportive ethos or policy (Cowie and Jennifer, 2007; Cowie and Smith, 2010). This means that it is an integrated element among practices and attitudes related to the improvement of relationships and the well-being of the entire educational community. Moreover, the degree to which students and teachers know about the peer support available in their school is important. In addition, the active backing of the head teacher or those involved in the school's management contributes to the program's success (Cowie and Smith, 2010). School staff as well as families are able to be positive influences by spreading the word and motivating participation. Finally, the extent to which students are able to have control over the intervention, properly contributing to it by themselves, needs to be taken into account.

Peers are included in psychosocial treatment approaches for students with SEND, more specifically for children and adolescents with ASC or Attention-Deficit Hyperactivity Disorder (ADHD), who show difficulties with social interactions (De Boo and Prins, 2007; Schall and McDonough, 2010). These interventions allow for opportunities in which they can learn new social skills and/or how to appropriately apply them. Three types of approaches in which peers are involved have been distinguished (Cordier et al., 2018): peer involvement, peer proximity and peer mediation. Peer involvement entails participants facilitating each other's learning when receiving instruction on social skills and frequently implies peers with similar difficulties should work together in a group therapy context. Peer proximity means that a carefully selected peer with adequate skills is placed in proximity to the focus student, for example sitting near them in the classroom. Peer-mediated intervention (PMI) is a treatment approach in which peers are trained or directed by an adult to instruct and/or facilitate social interactions (Chan et al., 2009). Students support their peers with disabilities typically by modeling and reinforcing appropriate behavior (DiSalvo and Oswald, 2002). Consequently, of these three groups of approaches, only PMI can be considered an emotional peer supporting practice—more particularly befriending—as detailed in the description presented above, if specific conditions are met. Peers should be able to facilitate social interactions with students with SEND and not have a mere instructional task. Additionally, the intervention should not be directed, at least not completely, by adults. Finally, the goal of the support lies in its befriending character. It aims to improve peer relationships in the school and not at the treatment of an individual's disorder.

Reviews of studies on PMI have demonstrated positive results for students with ASC in terms of treatment (Chan et al., 2009), substantial improvement in social interactions (DiSalvo and Oswald, 2002) and advancement of social skills (Miller et al., 2014). However, DiSalvo and Oswald noted that the nature of social interaction improvements varied across participants and studies. A review on augmentative and alternative communication intervention research showed that interventions for students with SEND that incorporated peers (including PMI) had a positive effect on communication (Fisher and Shogren, 2012). Travers and Carter (2021) reviewed the effects of PMI on students without disabilities (i.e., peer supporters) and found benefits in various areas, including social impact (interaction, friendships with others), increased knowledge about disabilities and changes in self-perception. Cordier et al. (2018) searched for research on peer proximity/involvement/mediation for children with ADHD but did not find studies on peer-mediated intervention. No conclusions about the efficacy of these types of interventions to improve these children's social functioning could be drawn.

In summation, the reviews on PMI interventions are important contributions, but as they only partially fit into the group of emotional peer support, further synthesis is necessary. Moreover, to our knowledge no review is available on this specific area. Therefore, our purpose is to perform a systematic review that focuses on emotional peer support interventions in school contexts (kindergarten, primary, and secondary school) for students with SEND and facilitate knowledge about this type of evidence-based practice. This paper's research questions can be grouped together in three areas:

(1) Which types of peer support have been employed and what is their degree of success in terms of interpersonal outcomes between students, as well as results on the individual level for the SEND students and their peer supporters?
(2) How is the selection and training of the peer supporters completed? What is the level of participation of the students themselves in the intervention? Are they able to contribute to the intervention (e.g., by proposing activities, etc.), or has it been fully structured by adults? And finally, are there possibilities for further relationship building outside the usual settings or is peer interaction limited to a fixed structure (in terms of time, space and activities)?
(3) How is the adult support performed? Furthermore, do the school and families support the intervention and in which way?

## Methods

### Search Procedure

The present literature review was conducted according to the PRISMA guidelines for systematic reviews (Liberati et al., 2009). First, in November 2020, we searched the peer-reviewed scientific literature for which the following EBSCO databases were used: APA PsycInfo, ERIC, Academic Search Premier, Education Source, MEDLINE, Psychology and Behavioral Sciences Collection, APA PsychArticles, PSICODOC, APA PsycBooks, Teacher Reference Center, Humanities International Complete, and Open dissertations. Additionally, the databases PsyArXiv and Search Open Grey were employed in order to include gray literature. Key search terms were selected on the basis of Cowie and Wallace (2000) differentiation of models of peer support (i.e., counseling but not tutoring) in order to include only specific types of emotional support, with the addition of the more general term *peer support*. Two Boolean searches were conducted, the first using combinations of the following keywords: 1. *peer support* OR *peer counseling* OR *peer mediation*, 2. *disability* OR *disabilities* OR *disabled* OR *impairment* OR *impaired* OR *special needs*, 3. *school* OR *education* OR *k-12* OR *elementary* OR *kindergarten*. In the second search, keywords of the first series were substituted for 1. *befriending* OR *conflict resolution* OR *circle of friends* OR *telephone help*; the remaining keywords were the same. A filter was used for age in order to include only abstracts of studies with participants in childhood and adolescence.

**Figure 1:**
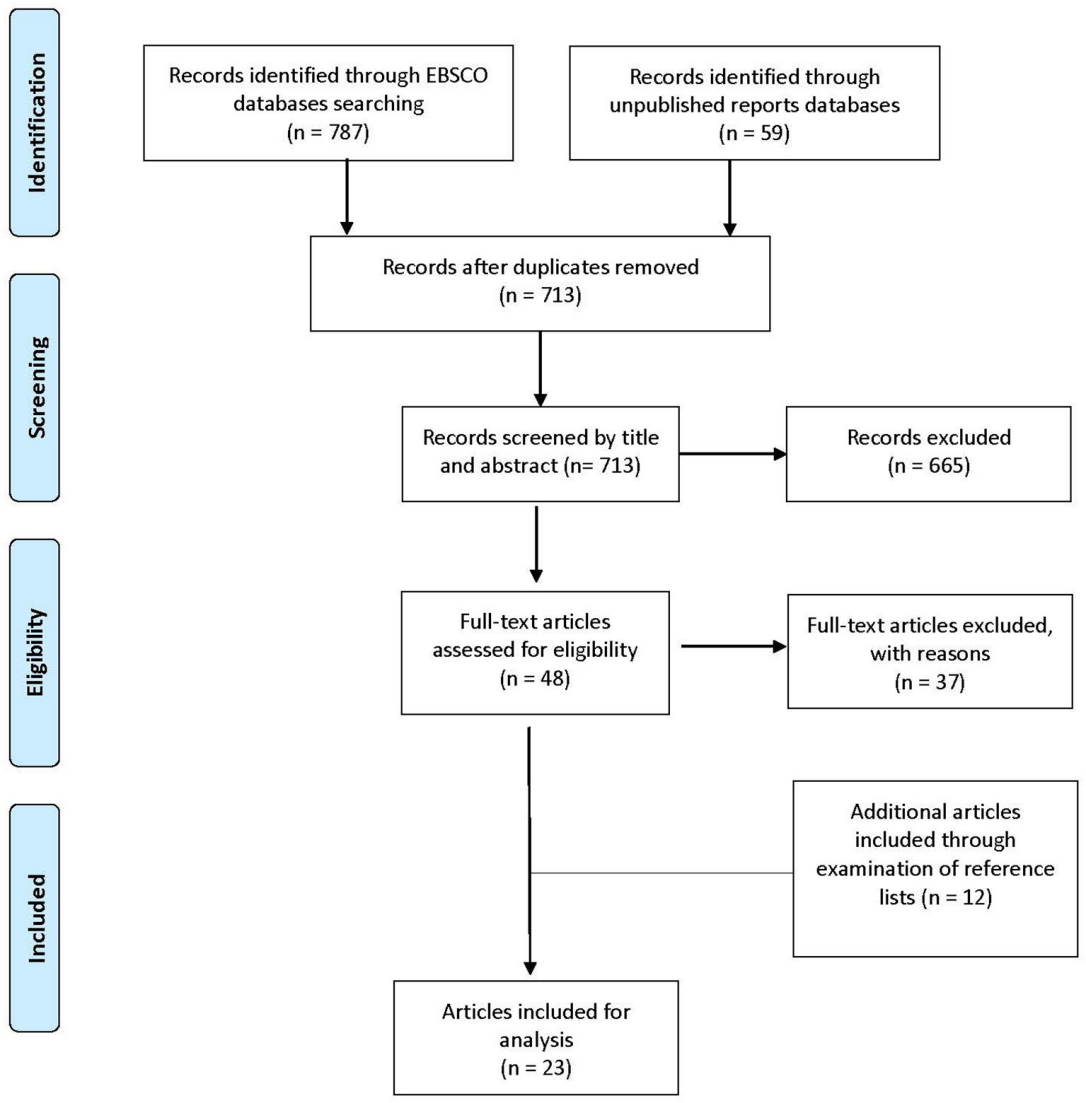
PRISMA flow diagram of study selection.

The PRISMA diagram Figure 1 shows the selection of included studies. Searches yielded 787 records in the EBSCO databases and 59 trials in the unpublished report databases, of which 133 were duplicates. After removing these duplicates, each of the 713 records was first screened by title and abstract by one of the authors. An intercoder was performed on 20 articles to ensure that all articles were identified correctly on the basis of the abstract, and consensus was reached on 95% of the sample. The initial disagreement on one paper was jointly reviewed until an agreement was obtained. A total of 665 papers, which included the total number of unpublished works, were excluded from further analysis. Of the remaining 48 records, the full text was obtained and assessed for eligibility applying the pre-established criteria with 11 papers meeting the criteria. Finally, the reference lists of the identified articles and relevant literature reviews were examined, and 12 additional studies were located, summing up to the total of 23 papers.

### Inclusion and Exclusion Criteria

Studies were included in this review if they met the following criteria: a. it involved an empirical examination of peer supporting actions, b. it took place in the school context: in kindergarten, primary or secondary school, c. participants were between 2 and 18 years of age, d. it was published in English or Spanish. There was no restriction related to the year of publication. Unpublished dissertations were excluded from the review, as they were not available online. The present review is not expected to be influenced by this since Vickers and Smith (2000) showed that the inclusion of unpublished dissertations rarely affects the conclusions of a review.

A paper was included only if the intervention involved one of the three types of emotional peer support of befriending, mediation or counseling. Practices that included didactic instruction, for example academic peer support as peer tutoring, or only physical assistance from peers to students with physical disabilities were excluded. Finally, interventions in which adults, e.g., teachers or therapists, were constantly involved in the process in a directive role by giving instructions when peers interacted were also excluded. Adults should mainly have an organizing and supervising role in the described practices.

## Results

The 23 papers that were included in the present review focused on four different types of emotional peer support. Nine articles involved a circle of friends (CoF) intervention, eight papers reported a type of peer buddying, four studies focused on peer networks and two articles portrayed social club interventions. Therefore, all papers document befriending interventions, and no articles were found on peer mediation or counseling-based support. The intervention procedures that were reported in these papers are briefly described below. In Table 1, data on participants, research methods and outcomes of each study are shown.

**Table 1:** Features of studies on emotional peer support interventions for students with SEND.

**References, Country**	**Educational level Participants: focus students, peer supporters, adult facilitators**	**Research design, methods, and measures**	**Peer and self-esteem related outcomes**
**Circle of friends**			
Bowen (2010), UK	Secondary education Focus student: Boy (12 yrs.) with visual impairment Peers in CoF: 7 children (not further specified) Adult facilitator: classroom teacher	Design: Pre-post and follow-up evaluation Method: questionnaire Measures: self-esteem, locus of control (focus student)	- Increased self-esteem, improved locus of control
Frederickson and Turner (2003), UK	Primary school Focus students: 20 students in different classrooms (19 boys, 1 girl, age 6–12 yrs.; Grades 1–5) with special educational needs (emotional and behavioral difficulties; learning difficulties). Peers in CoF: 4 to 8 classroom peers in each CoF (number of boys in each CoF, M = 3.47, girls M = 2.94); 19 CoF. Adult facilitators: educational psychologist (from outside school), graduate students in educational psychology, classroom teachers, specialist teacher/support assistant.	Design: Two-phase small scale evaluation. Phase 1: between-groups pre-post design; Phase 2: within-subjects design. Phase 1 (CoF set up for 10 focus students; 10 focus students served as control group). CoF led by graduate students. Phase 2: (CoF created for 9 focus students of control group in Phase 1). CoF led by school staff. Method: questionnaires Measures: Social acceptance/inclusion, social rejection of focus child. focus child's scholastic, athletic competences, physical appearance, behavioral conduct, social acceptance (self-perception by focus student, teacher's ratings). Global self-worth (focus student). Perceptions of classroom learning environment (all students in classroom).	- Improvement of social acceptance of the focus students by the peers in their classrooms; although more reduced to peers in CoF in Phase 1 - Increase of focus children's perceptions of self-worth participating in Phase 2 - No changes in perceptions of social acceptance (evaluated by focus students), behavioral conduct (rated by focus students, teachers) - No changes in perceptions of classroom's ethos (by whole class)
Frederickson et al. (2005), UK	Primary school Focus students: 14 students in different classrooms (11 boys, 3 girls; age between 6 and 11 yrs., Year 2–6) with learning difficulties (7), emotional and behavioral difficulties (6) and ASC (1) Peers in CoF: 6 to 8 classroom peers in each CoF; 14 CoF. Adult facilitators: assistant educational psychologists, classroom teacher (or other school staff) as participant observer	Design: Baseline (Time 1, before whole-class meeting) -intervention (Time 2, 3–5 days after whole-class meeting) -follow-up (Time 3, 1 week after circle meetings; Time 4, 1 term afterwards, only 7 CoF). Method: questionnaires Measures: Sociometric measure: acceptance, rejection of focus students Peer nominations for positive behavior (cooperating, leading) and negative behavior (disrupting, fights, bullying, victim of bullying)	- Increase of acceptance and reduction of rejection by full group of students in the class after the whole-class meeting - Weekly circle meetings did not produce further improvements in terms of acceptance and decrease of rejection, not for the whole class, and neither for students in CoF - No changes found in peer ratings of focus student's pos itive nor negative behavior -Exception of one CoF involving student with ASC, with continued improvement of acceptance and rejection scores, as well as a decrease in ratings of disruptive behavior.
Gus (2000), UK	Secondary school Focus student: boy w/ ASC (Year 10) Peers: whole classroom Adult facilitators: classroom teacher, external educational psychologist	Design: Case study. Qualitative evaluation 23 weeks after information session. Method and measures: Questionnaire (classroom peers)	- Classroom peers changed their attitude (more sympathetic, patient, understanding his feelings), as well as their behavior toward focus child (allowing him to join in, trying more to talk to him, telling others not to treat him badly).
Kalyva and Avramidis (2005), UK	Preschool Focus students: 3 boys w/ ASC (age 3/4 yrs.) Peers in CoF: 15 girls, 10 boys from same classes, typical development, similar age as focus students; 3 CoF. Adult facilitators: classroom teachers, therapist	Design: Baseline-intervention-follow-up evaluation. Comparison with control group (2 boys w/ASC, age 3–4 yrs.) Method: Observation Measures: 1. Responses to initiatives by peers to make contact, 2. Contact initiation attempts (by the focus child)	Focus children in comparison to control group showed: - Significant increase of successful responses and initiations of contact, - Significant decrease of unsuccessful responses and initiations of contact. Differences maintained during follow-up (2 months after intervention).
O'Connor (2016), Ireland	Primary school Focus student: boy w/ ASC (Asperger Syndrome, age 10 yrs.) Peers in CoF: children of same class (unspecified) Adult facilitator: classroom teacher	Design: Pre-post evaluation Methods and measures: 1. Observation. Behavior of focus child. 2. Questionnaires. Sense of belonging in school (focus child). Sociometric status (Peers in CoF)	- More contact between focus child and class group, higher quality of contact. - Increase of successful social initiations by focus child - Increase of focus child's feelings of social acceptance - Increased willingness of 70% by peers to work with focus child Peer group 80% more likely to accept behaviors shown by focus child - Outside school activities: invitation to 2 birthday parties, going to cinema - Peers in CoF also showed acceptance of other students (no ASC) who had been socially excluded
Owen-DeSchryver et al. (2008), USA	Primary school Focus students: 3 students with ASC (age 7, 7 and 10 years; grades 2 and 4). Peers in CoF: 7 girls, 3 boys from same classes, typical development; 3 CoF. One CoF of 2 boys substituted by CoF of 3 girls. Adult facilitators: Researchers	Design: Baseline-intervention-post-intervention evaluation (6 months). Method: Observation. Multiple sessions during lunchtime and recess Measures: For trained peers and untrained peers: 1. Social initiations toward focus student, 2. Responses to initiations made by focus student. For focus students: 1. Social initiations toward peers, responses to social initiations by peers. Both initiations as responses defined as positive social behavior.	- Increase of social initiations by both trained and untrained peers toward focus student, and of responses to initiations by focus student - Increase of social initiations and responses by focus children toward peers - One group of 2 male trained peers did not show an increase of social interactions, substituting group of 3 girls did show increase.
Schlieder et al. (2014), USA	Secondary school Focus students: w/ ASC, further unspecified Peers in CoF: unspecified, number of CoF unspecified but > 5 Adult facilitators: school personnel working with special needs students	Design: Multi-site collective case study. Qualitative evaluation. Method and measures: (Phone) interviews w/ (a) group facilitators, (b) parents of focus students, (c) community partnering agency program directors. Comparison of perspectives in data analysis. Use of measures to increase trustworthiness such as triangulation, member checking.	- Peer acceptance and less fear of classmates toward focus students, increased interaction, friendships - Peers in CoF showed increase of empathy, understanding of classmates w/ ASC and other disabilities, as well as to students outside CoF - In settings outside school (without adult facilitators), peer acceptance generalized as seen in outside activities (e.g., birthday parties, movies, gaming clubs)
Whitaker et al. (1998), UK	Primary and secondary school Focus students: 7 students w/ ASC (Years 3 to 10). Peers in CoF: 52 classroom peers; 7 CoF. Adult facilitators: classroom teachers, member Autism Outreach Team	Design: Qualitative evaluation. Repeated measures during intervention. Methods and measures: Interviews (focus children, school staff responsible for CoF, parents, other school staff). Questionnaires followed by discussion (CoF peers). Self-esteem measure (CoF peers).	- Improved quality and quantity of contacts between focus child and peer group - Reduced anxiety in focus children - Perception of focus children mainly as recipients of help, more than participating in a relationship in which both parts are equally supportive. - Only 3 CoF peers perceived focus child as a friend. Few outside school activities (one focus child received invitation to visit home of CoF peer, one focus child visited at home). - Increased levels of empathy and improved understanding in CoF peers, improved group participation, more CoF peers showed enhanced self-esteem in comparison to non-involved classmates over same period.
**Peer buddying**			
Artiles et al. (2016), Spain	Secondary school Focus students: School 1 (no peer support), Classroom 1: 7 students (age between 14 and 17 yrs.). School 2 (with peer support program). Classroom 2: 6 students (age between 17 and 20 yrs.). Classroom 3: 6 students (age between 15 and 20 yrs.). Various disabilities, including Down Syndrome, labeled as intellectual disability and ASC (except for classroom 3). Peer buddies: unspecified Adult facilitators: Special education teachers Classroom 2: teachers	Design: Multiple case study Method and measures: 1.Observation during break time Type of communication (oral, -type of- gestures, no interaction), transmitters and receivers (classmates, peers in school, teachers) 2.Semi-structured interviews with focus students Interactions during break time	For students from School 1 (without peer support) moments of no interaction (68%) during break time were more frequent than those of interaction. They mostly interacted with their classmates (77%) and very little with teachers (4%). The opposite happened for students from School 2: Classroom 2 interacted during 63% of the time observed, with other peers (44%), teachers (32%), classmates (24%); Classroom 3 interacted during 83% of the time, with other peers (21%), teachers (24%), classmates (55%). In School 2, teachers supervised interactions during break time, in School 1 they did not. Overall, interactions were close and without tension, frictions and anger were very infrequent.
Carter et al. (2001), USA	Secondary school Focus students: 109 students with severe disabilities, including ASC (73 boys, 36 girls), age between 13 and 20 yrs., 10th-12th grade. Peer buddies: 30 students (26 girls, 4 boys), age between 14 and 18 yrs., 10th-12th grade. Adult facilitators: Special and general education teachers, with assistance from researchers.	Design: Pre-test-post-test Method: Structured questionnaire for peer buddies and non-volunteers (*n* = 30; 26 girls, 4 boys; age between 14 and 18 yrs., 10th-12th grade). Measures: SDQ (Haring et al., 1983). - Social willingness to interact with students w/ disabilities - Knowledge of people w/ disabilities - Affect - Prior contact	Pre-test: peer buddies reported more social willingness and more prior contact with persons with disabilities than the non-volunteers. No differences were found in knowledge and feelings toward individuals with disabilities. Post-test: After participating one semester in the Program, peer buddies scored higher on social willingness, knowledge and prior contact. Additionally, a positive correlation was found between prior contact and social willingness. No changes between pre- and post-test-scores of the non-volunteers were observed.
Copeland et al. (2004), USA	Secondary school Focus students: 152 students with moderate/severe disability including ASC, limited communication skills (36% girls; age between 14 and 20 yrs.) Peer buddies: In study, 32 out of 53 participating students in the Program at least one semester (78% girls, 78% seniors), age between 16 and 18 yrs. Adult facilitators: Same as Carter, Hughes, Copeland & Breen (2001)	Design: qualitative research Method: 6 focus groups (2 to 11 participants) Measures: Perceptions of - Learning about Program - Peer buddy role - Benefits for themselves and others in school - Improvement of Program	- Actions of peer buddies to increase participation of students with disabilities in general education: enabling opportunities for interaction, advocating for students, modeling acceptance for other peers, increasing their own knowledge and skills, adjustment to friendship instead of teaching role - Perceptions of benefits for themselves (knowledge, attitudes, friendships, feelings of accomplishment), focus students (functional academic skills, social interaction), other students (opportunities for interaction, increased awareness of disabilities), teachers (assistance)
Copeland et al. (2002), USA	Secondary school Adult facilitators: special education teachers	Design: qualitative research Method: open-ended questionnaire for general (*n* = 13) and special education teachers (*n* = 13). Minimum experience in Program ≥ 1 yr.; most teachers ≥ 4 yrs. Measures: perceptions on: - Benefits for students w/ disabilities - Benefits for general education students - Challenges in implementation - Recommendations	- Benefits for students with disabilities. Special education teachers emphasized social-related benefits (increased interaction opportunities with peers and age-appropriate social skills acquisition. general education teachers primarily mentioned academic or functional skills related benefits. All teachers reported benefits in terms of establishing positive relationships (including friendships), enhanced personal growth
			(self-confidence). Additionally, peer buddy assistance is less disruptive in the classroom, therefore does not draw attention to focus student (marking them as “different”). Benefits for general education students. Socializing opportunities, students with disabilities are also positive role models, increased diversity.
Hughes et al. (2001), USA	Secondary school Focus students: 200 students (34% female) w/ severe disabilities (e.g., mental retardation, multiple disabilities, physical disabilities) participating in program Peer buddies: 115 students (82% female, 74% in grade 12) of 169 (83% female) students participating in program (10th-12th grade). Participation in Program: 50% participated ≥4 months; 50% ≥8 months (max. 2 yrs.) Adult facilitators: Same as Carter et al. (2001)	Design: qualitative research Method: open-ended questionnaire for peer buddies Measures: Attitudes toward focus students Benefits from interaction Type of activities Contributions made to focus students Suggestions for maintenance and improvement of program	Perceptions of peer buddies: - Positive attitudes toward peers w/ disabilities - Perceiving more similarities than differences (especially related to needs, desires and feelings) - Benefits for themselves (personal growth, friendships, knowledge about, strategies for interaction with people w/ disabilities) - Benefits for focus students: 1. Helping focus students learn skills (e.g., functional life skills, employment training skills) 2. Befriending, promoting social interaction, and acceptance
Hughes et al. (2002), USA	Secondary school Focus students: 1 male student, 4 female students w/ mental retardation, autism, language impairment (severe disabilities; age 15–22 yrs.). Peer buddies: 12 students (7 girls, 5 boys; 10th-12th grade) Adult facilitators: Classroom teachers. Intervention sessions: research team (graduate students)	Design: multiple baseline design across participants Method: Observation Measures: - Social interaction - Quality of interaction - Reciprocity of initiation of social interaction - Exhibition of communication behaviors - Type of conversational topics (e.g., peers, school events, jokes, movies)	- Increase of engagement of social interaction, as well as quality and reciprocity of students' interactions - Increase in range of communicative behaviors (focus students) - Increase in variety of topics discussed in conversations by focus students and peer buddies
Staub et al. (1996), USA	Secondary school Focus students: 3 female, one male students with moderate and severe disabilities (age 13–14 yrs., 7th and 8th grade). Student aides: 31 students (25 girls, six boys) in grades 7, 8 or 9). 7 student aides had mild special education needs. Adult facilitators: General and special education teachers, school administrators (principal).	Design: Case study Qualitative research. Methods and measures: 1. Observation of focus students, in classroom and during lunch time. Observations of behaviors, interactions and interpretations, judgments of these. 2. Semi-structured interviews with student aides, teachers, special education assistant, focus students and their parents. Including questions on inclusion of students w/ disabilities, on student aide program and its outcomes.	Focus students: increased independence and academic skills, behavioral changes, and expanded socialization with typically developing peers (including developing friendships). Student aides: increased socialization, acknowledgment by school community leading to improved self-esteem, increased understanding and appreciation for persons with disabilities, enhanced patience (for some students only), being more responsible.
Whitaker (2004), UK	Primary school Focus students: 9 male students, one female student (age between 6 and 7 yrs.), with moderate/severe ASC and limited expressive language. Peer supporterss: 10 students from Year 6. Adult facilitators: Experienced learning support assistant, nursery nurse.	Design: Qualitative study Methods and measures: 1. Observation. Joint attention behaviors, communication by focus student, shared play. 2. Semi-structured interviews with peer tutors. Perceptions of experience 3. Semi-structured interview with mother of peer tutor. Benefits and disadvantages of child's involvement.	- Increase of shared play Very little increase of communication of requests, communication for other purposes rare - No change in level of joint attention behaviors. - Although focus students showed to be enjoying activities, awareness of a shared experience was more difficult to detect. However, when these moments of connection happened, rated by peer supporters as best part of their work.
			Most difficult were moments of not feeling acknowledged or rejected. - Classmates were perceived as overall supportive.
**Peer network**			
Gardner et al. (2014), USA	Secondary school Focus students: 2 male students w/ ASC (age 18 yrs., 12th grade; 14 yrs., 9th grade) Peer supporters in 2 networks: 3 students (1 girl, 2 boys, 11th−12th grade) 3 students (3 girls, 10th−12th grade) Adult facilitators: special education paraprofessional and special education teacher (received training), research team	Design: Multiple baseline design across participants. Method: Observation Measures: Social interaction (communicative behaviors), social engagement, specific chosen communication skill for each focus student, support behaviors by peer supporters and adult facilitator, interaction quality, proximity.	- Increases in social engagement and peer interactions. - Medium quality of 18 yr. old focus student's interactions, lower quality of 14 yr. old's interactions. - Decrease of social interactions when peer network was withdrawn temporarily. - Network members considered each other friends. However, when no meeting was held, peers spend time with other people, on other activities.
Haring and Breen (1992), USA	Secondary school Focus students: 2 male students (age 13 yrs., 8th grade) w/ moderate-severe disabilities (ASC diagnosis for 1 student). Peer supporters in two networks: 4 non-disabled students (2 girls, 2 boys, age 12 and 13 yrs.) 5 non-disabled students (5 boys, age 13 yrs.) Adult facilitators: Unspecified.	Design: Multiple baseline design across participants. Methods and measures: 1. Observation. Social interactions (i.e., initiations and response), appropriate social responding, interactions outside of school (including weekends). 2. Qualitative self-reports. Peers' ratings of relationship w/ focus student, satisfaction w/ program. Degree of satisfaction of focus student with peer network.	- Increase of social interactions between network members. - Increase of appropriate responding by focus students - More positive perceptions of focus students Increase of descriptions of network members as friends - Interactions outside school (e.g., trips to the mall, beach), 12 times for one focus student, 5 times for the other (Baseline: no interactions outside school.)
Harrell et al. (1997), USA	Primary school Focus students: 3 students (2 boys, 1 girl) w/ ASC, communication difficulties (age 6–7 yrs., 1st grade) Peer supporters in 3 networks: 5 students w/ typical development (1st grade) in each network Adult facilitator: Researcher	Design: Multiple baseline design across settings nested within multiple baseline across participants. Methods: Observation, expressive behavior recording, computerized data collection, friendship and peer nomination scales. Measures: Social interaction duration Peer acceptance of focus student Inappropriate behaviors (focus student) Use of augmentative communication system (focus student, trained, and untrained peers) Frequency of expressive verbalizations (focus student)	- Increase of social interaction time across the various settings - Increased acceptance by peers (“like to play with”) Minimal exhibition of disruptive behaviors - Increase of augmentative communication systems by focus students and network peers in several settings - Increased expressive language for 2 students
Hochman et al. (2015), USA	Secondary school Focus students: 4 male students w/ ASC (age between 15 and 17 yrs., 9th−11th grade). Peer supporters in 4 networks: 9 female students and 2 boys (age between 16 and 18 yrs.) were trained; finally 7 girls and 2 boys participated in networks. Adult facilitators: one special educator and two paraprofessionals (received training), research team.	Design: Multiple baseline design across participants. Method and measures: 1. Observation. Social interactions (communicative behaviors), social engagement, specific chosen social/communication skill for each focus student, support behavior by adult facilitator, proximity.	- Increase of social interactions and social engagement during network meetings, but only remained high for one focus student on non-network days. - Modest gains of specific social/ communication skill for 3 students, high improvement for 1 student - Increase of proximity to peers supporters in network, as well as other peers (without disabilities).
		2. Questionnaires with Likert-type and open-ended questions. Focus students, their parents, peer supporters and facilitators. Questions on friendships and well-being (focus student), network experience and perceived outcomes.	- Positive perceptions of intervention by all agents. More interactions and friendships among peers w/ and without disabilities observed by adult facilitators. Peer partners noticed that focus students were interacting more with other peers outside network meetings. All focus students named peer supporters as friends; also peer partners considered student w/ ASC to be a friend.
**Social lunch clubs**			
Koegel L. K. et al. (2012), USA	Primary school; summer day camp Focus students: 3 students w/ ASC (boy 9 yrs., 3rd grade; boy 10 yrs., 5th grade; girl 12 yrs., 6th grade). Peer supporters in 3 clubs: 6–10 typically developing students in same grades Adult facilitators: university students	Design: Repeated measures baseline experimental design. Method: observation Measures: 1. Time engaged with peers (i.e., remaining in club area and interacting with peers) 2. Verbal initiations toward peers (questions, comments, activity directions)	- Increase of engagement with peers during club meetings. Increase of verbal initiations during club meetings. Average number of initiations was lower for focus students than for peers. However, on some sessions, all three children reached their peers' level.
Koegel R. L. et al. (2012), USA	Primary school, middle school Focus students: 3 male students w/ ASC (student 1: 13 yrs., 8th grade; student 2: 11 yrs., first school 6th grade, second and third school 7th grade; student 3: 14 yrs., 8th grade. Peer supporters in 3 clubs: unspecified Adult facilitators: unspecified	Design: repeated measures baseline experimental design. Method: observation Measures: 1. Time engaged with peers (i.e., remaining in club area and interacting with peers) 2. Verbal initiations toward peers (questions, comments, activity directions)	- Increase of engagement with peers during club meetings. Before intervention, focus students did not at all or nearly not engage with peers, although many clubs were available to them. - Increase of verbal initiations during club meetings. - Student 1 received multiple invitations to hang out/birthday parties from peers in same club. - Student 2 showed some generalization of engagement /and initiations to his second and third school.

### Description of Emotional Peer Support Interventions

#### Circle of Friends

With respect to the procedure of CoF, seven papers (Whitaker et al., 1998; Frederickson and Turner, 2003; Frederickson et al., 2005; Kalyva and Avramidis, 2005; Bowen, 2010; Schlieder et al., 2014; O'Connor, 2016) refer to the procedure as described by Taylor (1996; 1997; or to Whitaker et al., 1998 who in turn cites Taylor). According to Taylor, establishing a CoF involves several key components or steps. First, the commitment of the school is necessary for resources, and permissions from parents or guardians also need to be obtained. Second, a discussion is held with the entire class, but without the focus student, which is usually led by an external agent (e.g., educational psychologist or school staff member with no direct relationship to the class) and with the teacher being present. The key element of this session is a participative group discussion of the focus child's strengths and difficulties, in addition to a talk about friendship, the lack of it and the effects of that absence on feelings and behavior. At the end of the meeting, volunteers for a support group are sought for. Next, a group of six to eight students are selected for the CoF and in the first meeting with the focus child and the adult facilitator, goals and strategies are identified for the following week. Hereafer, the CoF meets weekly, again with help from the adult facilitator, to evaluate the progress and to make practical plans for the following week. These weekly meetings continue for about 6–10 weeks.

Of the seven reports, five had introduced small changes in the procedure of the intervention. Frederickson et al. (2005) changed the class meeting of one of the CoF into a session in which social interaction and communication difficulties of ASC children were explained, and how these difficulties affect their behavior. The goal was to make children aware of the low probability that the behavior of the focus student would change. Schlieder et al. (2014) followed the description by Taylor, although they also report having included specific CoF lessons about autism and motivation for positive interaction (without further detail). Kalyva and Avramidis's (2005) CoF implementation took place in preschool, therefore, it was adapted to that educational level. First, the peers for the CoF were selected by the teacher. She explained (in absence of the focus child) that the circle's goal was to help their classmate to learn how to ask other children to play. In the circle sessions, the teacher gave directions to the children for an imitation activity with toys. Bowen (2010) reported a CoF intervention for a boy with visual impairment, but in her rather short description a class discussion was not mentioned. Exceptionally, an exact reference to Taylor's model (or Whitaker) was not provided. Finally, O'Connor (2016) does not mention a whole-class discussion either in her report.

The last two papers involve a somewhat different procedure of the CoF. While Gus (2000) reports a regular application of only the first two steps, she also included a talk about autism in the class session as a replacement for the talk about friendships. A third step involved a feedback session 1 week later with the whole class, in which students answered questions about what they learnt and what they could do to support the focus student. No circle of peers was established hereafter; every decision was left in the hands of the class. In Owen-DeSchryver et al's. (2008) intervention, firstly peers were selected for a “training.” The first phase of this training consisted of either a book reading and a discussion about a child with autism (second graders) or a friendship awareness activity (fourth graders). In a second phase, a discussion about the strengths and weaknesses of the child with ASC took place, while during a third phase the CoF participated in a guided discussion in which concrete information and strategies for interaction were presented. The focus children were unaware of the peer training.

#### Peer Buddies

Eight papers involved a type of peer buddying, all in secondary education except for one intervention in primary school. Staub et al. (1996) described a “student aide program” in a junior high school in the USA. In every class period, a different student aide helped one of the focus students. Their tasks were varied: monitoring the focus student's behavior, helping with academic work and daily activities, and offering companionship (befriending). Student aides received training (unspecified in the paper) and obtained course credit as well as a course grade.

Artiles et al. (2016) carried out a study on the “Friend Project” implemented in a secondary school. Focus students were in special education classrooms in mainstream schools. Peer supporters accompanied focus students during recess to increase their participation and to help them acquire the skills they were working on in class. They also went with the focus students to complementary activities during break time, such as choir, dance and workshops. Peer supporters volunteered to participate and they received training (unspecified in the paper). Artiles et al. compared this school, in which teachers supervised interactions between students during recess, with a second school without a project nor teacher supervision.

Whitaker (2004) portrayed an intervention in a primary school, in which so-called “peer tutors” met focus students individually in their own special education unit in the school for play sessions on a weekly basis during lesson time (20–30 min). Supervising adults adopted a low-key role, giving only general advice when appropriate. The playing involved games and activities that were popular with the focus students. During the first 3–4 weeks, no instructions were given. Hereafter, peer supporters received one training session on how to support interaction (e.g., getting close, slow, and simple talking).

Implementation of the Peer Buddy-Program (Hughes et al., 1999) in several high schools in the Metropolitan-Nashville school district in the USA was evaluated and documented in five papers included in this review (Carter et al., 2001; Hughes et al., 2001; Copeland et al., 2002, 2004). The program consisted in providing support to peers with disabilities for at least one class period each day (50–90 min), in both in-school (e.g., classrooms, libraries, school cafeterias) and out-of-school settings (e.g., stores, shopping malls, employment sites). Activities were instructional (e.g., academic support, job training skills) and non-instructional (e.g., “hanging out,” participation in sports). Teachers chose both the activities and the settings for the students. Peer buddies were paired one-on-one with focus students or with several partners. For participation in the program, students earned course credit and they were able to participate for one or more semesters. Before starting, they received an orientation session in which several issues were discussed, including course expectations, disability awareness, communication strategies, dealing with inappropriate behavior and activity suggestions. They also obtained a “Peer Buddy Manual” with information about disability issues and interaction strategies.

The peer buddy intervention described by Hughes et al. (2002) seems to be related to the Peer Buddy-Program, however it is described in an independent way. Each day, classroom teachers assigned responsibilities to the peer buddies but they did not instruct them to socially interact with the focus students. Peer buddies aided focus students with assignments in their classes, or during extracurricular activities. In specific intervention sessions (led by the research team), peer buddies were asked to “hang out like a friend with the focus student,” engaging in a leisure activity with the focus students in three settings: classroom, cafeteria during lunch and gymnasium during physical education. Peer buddies were told it was not necessary to teach the focus student anything and no further instructions were given. Activities (e.g., playing games, walking laps) were drawn from a pool developed for each focus student and varied daily. Activity pools were created based on the observation of the students' leisure activities (by the research team) and classroom teachers' suggestions. Peer buddies were rotated across participants, with a total of 5–8 buddies helping one focus student. Participating students received a training course which was not further described and obtained course credits.

#### Peer Networks

A total of four articles described a peer network or “social network” intervention. Haring and Breen documented probably the first implementation in a junior high school in 1992. During transition periods one peer supporter met with the focus student, for example to hang out in the hall or walk to the next class. The group of peers in a network would also meet for lunch with the focus student on some days. A schedule of assigned transition periods was made by adult facilitators and provided to the peer supporters, who were also allowed to hang out freely with the focus student. Network meetings took place once a week, with the presence of an adult facilitator. These meetings included a discussion of skill areas that required support, the development of appropriate interaction strategies as well as the adjustment of schedules and problem solving. The role of the peers was maximized in these activities. During a following “maintenance” phase, that occurred only for one focus student, network meetings (2x per month) involved only discussing extended friendship interactions (e.g., meeting on weekends) and problem solving. To set up the networks, special education teachers selected students who had some previous contact with the focus student. The selected students (1 or 2) recruited 2 to 4 close friends for participation. Throughout the network meetings peer supporters were taught about specific aspects of the intervention, for example how to model appropriate social responses to the focus student.

In Harrell et al.'s (1997) intervention in three elementary schools, peer networks were implemented over three settings: (1) academic games and cooperative learning activities during reading activities (one focus student) or language-art sessions (two focus students). (2) interaction and conversation during lunchtime in the lunchroom. (3) language and arts/computer games/recess activities on the playground or in the classroom. Network meetings took place one to three times per day for 20 min, on 3–4 days per week. The activities were selected by the first researcher and by teachers. Conversation topics during lunch were chosen beforehand by the students themselves. Peers were instructed to interact with the focus student via an augmentative communication system and participate in activities. All students were trained in the use of the augmentative communication system, consisting in one-to-one teaching sessions (18–21 sessions for each focus student). Also the peer supporters received training on several issues, including instruction about the use of the augmentative communication system, the use of social skills for interaction with the focus student, discussions of autism, qualities of a friendship and how the network could help the focus student make friends. Training for each network comprised of eight sessions for two weeks before the start of the peer network meetings. The focus students attended the last two sessions as well.

Hochman et al. (2015) worked with four peer networks in two high schools. Weekly meetings were held during lunch periods in the cafeteria and included eating, activities (e.g., board games), conversations on events in and outside of school, planning new activities and reporting interactions which took place outside the weekly meetings. Other peers (with or without disabilities) sometimes joined the group. Adult facilitators were present for at least 10% of the meetings and intervened sometimes, for example by facilitating interaction or mentioning new school events. Generalization probes took place once a week and on that day no meeting was held. Students could then sit with whom and wherever they wanted. At the beginning, an orientation meeting was held with the focus student and the peer supporters, in which network goals were discussed and ways to work toward social interactions were considered by the group. A peer buddy program was also present in one of the schools.

Network meetings in Gardner et al.'s (2014) intervention were held once or twice a week with the presence of an adult facilitator, who was aided by a research team member. During the meetings, peers participated in at least one shared activity, e.g., a conversation about school events, playing or teaching others to play a game. Specific social-related goals for each focus student were selected by the educators and addressed during 50–75% of the meetings. An orientation meeting of the same characteristics as reported by Hochman et al. (2015) was arranged at the beginning of the intervention.

#### Social Lunch Clubs

Finally, two interventions involved so-called “social lunch clubs.” Koegel L. K. et al. (2012) described their intervention as following. First, the focus student's favorite activities were determined in an interview with the child; parents were also consulted. Second, a club was created with the favorite activity as the club's theme. Club meetings happened twice per week during the lunchtime period for 15–30 min. At the start of each meeting, peers were invited to join the club (verbally or with a poster advertising the club). One or two adult facilitators conducted the activities: games and arts and crafts activities. In the club of the participating 12-year-old girl, the group was also led by peers and included planning and celebrating a party at her house.

Koegel R. L. et al. (2012) reported on a social club intervention in one primary and four middle schools, in which diverse ongoing clubs (academic activities, sports, arts, and crafts) were already available to students during lunchtime. A social club was created for each focus student around their perseverative interest and presented through announcements, flyers and notes to parents (just as for other clubs). The focus student's diagnosis was not disclosed to their social club partners. Peers were not informed that the club was created around the focus student's interests either. In both reported interventions, peers participated on a voluntary basis in the clubs, and did not receive previous training.

### Educational Level, Age, and School Type

In sum, more than half of the CoF interventions (*n* = 5) were carried out in primary education (one in preschool, three in secondary school), while all peer buddy interventions were implemented in secondary education except for one project taking place in a primary school. Five of the papers reporting on these last interventions involved a Peer Buddy Project with overlap in the participating schools of one school district. Furthermore, peer networks were also mostly implemented in high school (three against one in primary education), while social clubs were created in one middle and two primary schools.

In relation to age, it can be observed that in three CoF studies, the age range of the focus students was large (6–11, 6–12, 7–15 years). The remaining CoF interventions were set up for students of a particular age, which on the whole showed a varied set of ages up to 15 years (3/4, 7, 10, 12, 14/15 years). The ages of the CoF members were never specified, but they were mostly labeled as classroom peers, or of a similar age. Wide age ranges were also described in the peer buddy interventions, both for focus students and peer buddies, although they all included the period of adolescence (e.g., 14–17, 13–20 years). Ages of the participants in the remaining studies were again diverse, between 6 and 18 years, with peer supporters in the same grade or having a similar age, except for Whitaker's (2004) study in which peer supporters were 3–5 years older.

CoF were all applied in mainstream schools, one special school was also involved. Focus students had learning or emotional/ behavior difficulties (*n* = 2), visual impairment (*n* = 1), but in most cases (*n* = 7) the CoF were set up for students with ASC. Permanent support by an assistant in the classroom was reported in three papers. Focus students were described as victims of bullying in three occasions, however in four papers, descriptions or indirect references to poor peer relationships or isolation problems were found. In contrast with CoF interventions, focus students in the peer buddy, peer network and social club studies were mainly in self-contained classrooms (as reported in 12 out of 14 papers), in addition to receiving part of their classes in general education (all Peer Buddy Project papers (n = 5); social clubs, *n* = 1; peer networks, *n* = 3). Exceptions were two interventions taking place in mainstream education, and three reports in which some children and adolescents received education in special education (some receiving classes in general education) and others in mainstream schools. The focus students in all these described practices were students with ASC (12 papers), severe disabilities (*n* = 9) or moderate disabilities (*n* = 5). Explicit references were not given to these students being victims of bullying in any of the articles.

### Study Methods

The research included in this review comprised various types of designs such as a single case study or group experimental design. Investigators used diverse measurement tools, including, for example, interviews with various stakeholders, observation of children's behavior during recess, as well as peer nominations and self-esteem measures. To summarize common measurement aspects, 15 studies collected experimental data to measure impact, either by means of questionnaires (*n* = 5), observation (*n* = 8) or both (*n* = 2). In addition, 10 studies involved qualitative methodologies, performing interviews (n= 2), observations and interviews (*n* = 3), focus groups (*n* = 1), or collecting questionnaires (n= 6), on one occasion the collection of the questionnaire was followed by a discussion with the participating students.

### Outcomes for Focus Students and Peer Supporters

Interpersonal outcomes between students as well as results on the individual level for SEND students and peer supporters were analyzed. Overall, interpersonal results of the interventions were mainly positive for the focus students. In 19 of the total 23 papers an increase of social interaction with peers was found, also labeled as enhanced engagement, socialization, or shared play. Looking in more detail, in four studies focus students were found augmenting their proximity to peers, or their levels of successful initiations and responses of contact. On four occasions, enhanced quality of interaction was reported. An increase of communicative behaviors was found in six papers (including verbal initiations, expressive language, more variety of topics in conversations). Additionally, peers showed increased social acceptance of the focus students, as reported in six articles. On the individual level, focus students improved their self-esteem (*n* = 2), social skills (*n* = 3; students with ASC/severe disabilities), changed their locus of control (*n* = 1), increased their feelings of social acceptance (*n* = 1); reduced their anxiety (*n* = 1), challenging behavior (*n* = 2) and their needs for adult support (*n* = 1). Poor social outcomes for the focus students were reported more infrequently: no increase of interactions, e.g., during a particular period of the intervention (*n* = 3), decrease of interaction when peer network was withdrawn (*n* = 2), lower quality of interactions (*n* = 1) and unchanged perceptions of social acceptance (*n* = 1).

For the peer supporters, the interventions also mainly produced benefits. Apart from the increased interaction with the focus students, the reviewed studies showed: enhanced empathy and improved understanding, changing to positive attitudes toward classmates with disabilities, perceptions of similarities more than differences, thinking of focus students as positive role models, feelings of connection (with severe ASC students), increase of interaction strategies, communicative behavior (*n* = 2), and enhanced self-esteem and self-expression. Only few difficulties were found, which were in relation to engaging with the behavior of ASC students and the feeling of not being acknowledged by the peer with severe ASC (each of the cited effects was reported in one paper except where noted).

To analyze true “befriending effects” of the interventions, the papers were checked for information on peers sharing other spaces and activities than merely the planned group meetings (e.g., network, CoF), in addition to findings related directly to friendships. Most papers included limited information on these issues or offered data of an anecdotal nature. In 11 papers a reference to friendship as an outcome was made in terms of an increase of peers describing themselves as friends, or the enhanced creation of positive relationships that included friendships. However, these were general descriptions that gave no information allowing for a more detailed analysis on the extent of the friendships between peers. Nevertheless, six papers included data on the peers' involvement in other activities, mostly outside school: meetings on the weekend, the focus student receiving invitations to hang out, to birthday and holiday parties, shopping trips, gaming clubs and movies. Adults observed typical friend behavior; students exchanged phone numbers. An exception to the overall positive perception of friendship gains is the more precise report by Whitaker et al. (1998) on the effects of the implementation of seven CoF. Of the 40 involved peers, only three students considered the focus child to be their friend and invitations or visits home by the peers were scarce (two for two focus children).

### Description of Peer Supporters. Selection and Training

Peer supporters were children and adolescents with typical development, excluding the interventions reported by Staub et al. (1996) and Whitaker et al. (1998) in which they were peers with mild special educational needs or students in special education. Overall, multiple peers aided one focus student, except for the peers in the Buddy Project, who were involved in a one-to-one support format. Information about the gender of the peer supporters was only available in 11 papers. In three CoF studies with reported data, participation was equal between girls (*n* = 78) and boys (*n* = 79). Data from the three peer network interventions also showed similar numbers for both genders: girls (*n* = 13) and boys (*n* = 11). From the four papers with data on the “Peer Buddy Project,” the article by Hughes et al. (2001) was selected to analyze participant data, as it reported the highest number of participants and it was suspected there would be an overlap with figures from the other papers. A significant higher amount of volunteering girls (*n* = 144) than boys (*n* = 25) participated in the project. However, in the study by Staub et al. (1996) more boys (*n* = 25) than girls (*n* = 6) were selected. No data was found in relation to the social lunch club interventions.

The criteria that were used to select the peers for the CoF (*n* = 9) were emotional skills (*n* = 1), diversity in gender (*n* = 1), varied abilities (*n* = 3), adequate attendance, ability to make up schoolwork, compliance with teachers and willingness to participate (*n* = 1) or unspecified (*n* = 3). In the remaining study, no selection was performed, as the whole class participated in the circle of friends. For the peer buddy interventions (*n* = 4), selection was based on skills (*n* = 2), availability (*n* = 1), adequate grade point average (*n* = 1), attendance (*n* = 1), age (*n* = 2), presenting an application reference (*n* = 2), likely commitment (*n* = 1) or merely volunteering (n = 2). In addition, students received credits for participation (*n* = 2). The papers on the “Peer buddy project” were counted as a single study in this analysis. With respect to the peer networks (*n* = 4), selection criteria were skills (*n* = 3), previous contact with focus students (*n* = 1), dependable (*n* = 1), with a network of friends (*n* = 1), social status (*n* = 1), consistent school attendance (*n* = 1), compliance with teachers' requests (*n* = 1), merely volunteering (some students in one study). In one peer network study, peers recruited close friends for the intervention. Finally, participation in the social lunch clubs (*n* = 2) was only based on the students' interests. In sum, in most interventions peers were selected for the intervention, except for the social clubs and two other practices, in which the whole class participated, or in which involvement was purely voluntary. In part of the CoF interventions, children with varied abilities were selected, while for peer networks, the student's skills as well as having a social network were important. Partaking in the “Peer Buddy Project” was based on students showing interest and availability, although in two other peer buddy practices students' competences and likely commitment were taken into account.

Training of peer supporters was described in all interventions except for the social clubs. As explained earlier, in four of the reported CoF interventions, a whole- class session was held in which students learned about autism (*n* = 4) and positive interaction or interaction strategies (*n* = 3). In both the peer network and peer buddy interventions (*n* = 8; “Peer buddy project” papers considered as one), training involved building awareness of disabilities-autism (*n* = 2), social and communication skills (*n* = 5), use of an augmentative communication system (*n* = 1) or was not further specified (*n* = 2). Students in the “Peer buddy project” received a Peer buddy manual.

### Peers' Opportunities for Participation in the Interventions

#### Circle of Friends

How peers can contribute to the implementation of the CoF is included in Taylor (1996; 1996) description of the process, mostly in a more general way. Students were asked to participate in the definition of goals, evaluation of progress, identification of difficulties and the planning of ways to solve them, accompanied by an adult whose tasks have a primarily facilitating role. Nevertheless, the extent to which children participated in these tasks does not become clear, as it was not precisely documented in the papers.

Exceptions to this form of participation were found in the two reports. The practice of Gus (2000) consisted of two sessions in which the adult fulfilled a complete leading role; hereafter the task of supporting the focus student is left in the hands of their peers. More limited opportunities to participate seemed also evident in Owen-DeSchryver et al. (2008) intervention because of the accentuated guiding role of the adults, even though the children were asked to make their contributions in discussion-sessions.

#### Peer Buddies

In the primary school interventions (Whitaker, 2004; Artiles et al., 2016), children were just told to play as they were supervised by adults, they were not involved in organizing the intervention. Students' participation in planning tasks in Staub et al. (1996) intervention (secondary school) was not specified. In the Peer Buddy Program implementation in high school, students participated in a structured plan of leisure activities, however they also had opportunities to freely interact with the students.

#### Peer Networks

In meetings of peer networks (Haring and Breen, 1992; Gardner et al., 2014; Hochman et al., 2015), secondary school students were motivated to actively participate by the suggestion of activities or conversation topics. Adult facilitators were present for at least 10% of the meeting (Gardner et al., 2014; Hochman et al., 2015). However, Gardner et al. informed that early activities were mainly designed by the adult facilitators, and support behavior (encouraging focus student to interact with peers or vice versa, etc.) oscillated between 29 and 37%. Hochman et al. also reported that support behavior varied between groups (7–30%) and remained high in two networks. Peers were able to freely spend time with each other outside weekly meetings and outside the school setting. In Harrell et al. (1997) intervention in primary school, student participation seemed to be more reduced: activity selection was performed by adults. Nevertheless, conversation topics were selected by the children.

#### Social Clubs

Although club activities were conducted by adults, the researchers pointed at the high implication of the children themselves during the sessions, directing the group's activities and organizing meetings outside the club, at home.

### Adult Facilitators and Support by School and Families

Adult facilitators in the reviewed interventions were general education classroom teachers (cited 9 times), special education teachers, therapists, nursery nurses (*n* = 9), special education support assistants (*n* = 2), (assistant) educational psychologists (*n* = 3), school staff (*n* = 1), school administrators (*n* = 1), unspecified (*n* = 2: peer network (*n* = 1), social lunch club (*n* = 1).

In the nine reports on CoF, classroom teachers were involved in six studies, either alone (*n* = 2) or with other staff or external agents. With respect to Taylor (1997) recommendation to involve an external facilitator for the whole-class discussion while the class teacher is present, four interventions followed this suggestion by introducing an educational psychologist. General education classroom teachers were never involved in peer network interventions; networks were facilitated by either special educators or researchers. In three studies, only researchers acted as implementers (CoF = 1, peer network = 1, social lunch club = 1). The various adult facilitators cited in the reports on the “peer buddy project” (reported in five papers), were included only once in the analysis. From the full set of peer buddy interventions, special education workers were the only implementers on two occasions, classroom teachers were facilitators in three interventions (twice with other professionals).

In addition, papers were screened for information on the support that was received from the school as an institution as well as particular school staff, in addition to families. Only the articles that reported on this matter are cited below. Copeland et al.'s (2004) reported in their study that the high school students in the Peer Buddy Program perceived the general education context as unsupportive for the inclusion of SEND students caused by aspects such as lack of accommodations or differential expectations of educators. This fostered negative attitudes toward students with SEND by some teachers and students, affecting the program negatively. The authors also observed that in each school, typically, the promotion of the support system depended on only one adult; students might therefore not be aware of the possibilities for participation.

Hochman et al. (2015) emphasized the role of a teacher by reporting that getting the students involved, training them and keeping them in the program with ongoing support was much easier at the school where this was a very popular teacher, meaning that he connected very well with the students. These authors also comment that a peer buddy program was present in one of the collaborating schools, therefore showing that the peer network intervention was not a casual, isolated practice.

Staub et al. (1996) describes the participating school in their study as one in which best practices in education, striving for equality and cooperation, were common. For the CoF intervention, authors referred to the first step of setting up a circle (Taylor, 1997), in which it is necessary to check if the school is a place where the CoF can come to life and be given continued support. Although most evidently referring to a commitment from the school staff to provide the necessary resources (staff time, organization) and cited as such by Frederickson and Turner (2003), Frederickson et al. (2005), Schlieder et al. (2014). Whitaker et al. (1998) described the participating schools as having “an ethos compatible with the values underlying the ‘circles approach”' (p. 61).

Family participation was mentioned in the reports on the social club interventions. Koegel R. L. et al. (2012) also sent information to the families in order to encourage student participation in the clubs. In their second study (Koegel L. K. et al., 2012), a club session also took place at the focus child's house. In addition, Whitaker (2004) highlighted the important role of the parents and the support of their children in participating in the peer buddies project was extremely high.

## Discussion

The aim of this systematic review was to examine emotional peer support interventions for students with SEND. A limited number of studies were found evaluating this type of educational practice, all involving befriending interventions. The most documented practice was Circle of Friends, which already has a history of several decades, starting off as an intervention for the inclusion of adults with disabilities in their communities and for SEND students in mainstream schools (Sullivan, 2010). This was followed by the more recent peer buddying interventions such as the “Peer Buddy Project,” on which multiple papers were found including articles on peer networks and social club interventions. Empirical reports on peer conflict resolution or counseling-based support were not found. This finding might not be surprising as these system formats are usually not aimed at specific groups but rather at the general student population. Still, researchers could have collected data on the use that students with SEND make of these types of peer support, but this was not the case. In the future, it would be useful to know more about the peers who offer and receive counseling or conflict resolution support, and whether there are students with SEND among them.

The focus students in the reported interventions were mostly children and adolescents with ASC or, to a lesser extent, labeled as having severe or mild disabilities. Only in CoF interventions a reduced number of students with other (visual, emotional-behavioral) difficulties were included. Despite the existing literature on available psychosocial interventions for students with ADHD that include peers (see Cordier et al., 2018), in the present study no paper with explicit reference to participants with ADHD was found. CoF were mainly applied in general education schools and the participating peers in the interventions were classmates. On the contrary, most of the remaining interventions were performed in a fairly non-inclusive context, aiming at promoting interaction between students who received education in separate places (classrooms, buildings), and who only coincided in time and space on some occasions (one or two class periods, cafeteria, at recess). This proves to be a difficult task to be accomplished. As Copeland et al. (2004) point out, just being in proximity to peers who are in special education does not easily result in interaction. The lack of contact seems evident in those study settings, but also in the CoF studies, in which several focus students were described as having “isolation problems” or as suffering from negative interactions from their peers as victims of bullying. Nevertheless, outcomes for the targeted students of the interventions were overall positive with respect to several areas. In the majority of studies an increase of social interactions was found, in addition to other findings, though less reported, such as an increase of contact initiations and responses, proximity, communicative behaviors, quality of interaction, social acceptance by peers and improved self-esteem. Moreover, CoF seems to have been successful in addressing social difficulties or bullying experienced by students with SEND, especially ASC. However, the extent to which the reported increase of peer interactions happened for all students, and in each setting (classroom, school, outside immediate intervention context) remains uncertain. In Copeland et al. (2004) study for example, students declared that interaction with peers in special education was unlikely to occur unless a peer buddy (or a teacher) prepared the way for it.

Social skills improvement of students with ASC or severe disabilities was reported in a few studies. According to Hughes et al. (2002) this merely stems from interacting more with peers in mainstream education. They argue that for particular social skills, students with intellectual disabilities may need instruction, but when opportunities for interaction are provided, they are able to improve communicational behavior and gain conversational topics. Nevertheless, to obtain social acceptance from peers, an increase of social skills might not even be necessary. Based on the impact of their interventions, Frederickson et al. (Frederickson and Turner, 2003; Frederickson et al., 2005) stated that the CoF had resulted in a greater understanding of the classmate's difficulties by their peers rather than changing their behavior. They also observed that the weekly meetings of the CoF process did not produce any effects, while the whole-class meetings resulted to be essential for the intervention's outcome. The reason for this was the participation of all the students in the session. The CoF members already showed positive attitudes toward the focus child before the intervention, but other students in the classroom made a change after the meeting. In line with this stands the intervention by Gus (2000) who modified the CoF procedure by using only the whole-class meeting, in order to have all students participate in helping the child with ASC who had been socially excluded.

Outcomes for peer supporters were also mainly positive, showing benefits such as increased self-esteem and empathy, which are in accordance with earlier findings (Naylor and Cowie, 1999; Del Barrio et al., 2011). They also increased their interaction with the class or schoolmates with SEND. These interactions seem to have led to further bonding, reported by the authors through anecdotic evidence and general observations, resulting in an overall perception of friendship expansion. However, Whitaker et al. (1998) found less promising results and more precise research is needed.

Peer supporters were mainly typically developed children and adolescents, who received training when required for the intervention. Overall, boys and girls were found equally involved in these practices when a selection was performed by adults. This stands in contrast with the data from the Peer Buddy Program, whose requisites for participation were, above all, interest and availability and for which girls showed to be significantly more willing to volunteer than boys. This is in line with Cowie and Smith (2010), who claim that a frequently reported finding is that it is easier to recruit girls than boys for peer support. Boys might be afraid that the caring qualities they exhibit as peer supporters will not be considered manly (Naylor and Cowie, 1999). This points at the need for a gender approach in school ethos perception, in which boys and girls are equally responsible for mutual care.

Student possibility for real participation in the intervention has been underlined as an important success factor. In relation to the CoF, Taylor (1997) points out that when students are asked for their opinions and feelings, power is being redistributed from the adult to the children. Taylor argues that the way in which the teachers handle this, in combination with the school ethos, is crucial for the effectiveness of the intervention. However, in the reviewed papers on CoF interventions it is not clear to which extent the children were able to participate in the various tasks (e.g., in the weekly meetings). In other support practices, students contributed by suggesting activities or conversation topics, which in several cases, especially the social clubs, this was highly successful. Although some of the reports pointed at adults still playing an important role in designing tasks or supervising interactions, other studies succeeded in making students more in charge of the activities. Haring and Breen (1992) conclude that allowing for enhanced peer control instead of adult mediation may contribute to improved, more solid and long-lasting peer relationships.

On the other hand, student involvement in the proper organization of the systems (e.g., recruitment and selection processes) was hardly mentioned. Only in one peer network study did students recruit close friends to join the intervention. As a final point, students usually do not merely interact in the structured context of school, especially not adolescents. Therefore, the possibilities to socialize outside the established frame are important for genuine peer inclusion, and in several practices of peer buddying and peer networks opportunities to do so were given.

Information on support from school as an institution was scarce in the reviewed studies. However, it is a relevant factor to take into account when analyzing the effectiveness of peer support systems (Cowie and Smith, 2010). In the reviewed papers only some CoF implementers referred to commitment of the school's administrators, and even fewer remarks were found on the school ethos. An exception to this was Copeland et al.'s (2004) analysis of student perception on how negative attitudes toward students with SEND challenged the peer buddy's program, next to their observation on the significance of depending on a single adult for the promotion of the support system. The authors explain that the school's management and teaching staff might not be aware of the benefits of a support scheme for and by peers, and recommend promoting information about such programs.

Furthermore, the involvement of the class teacher is highly relevant, as they are role models for their students. In peer support systems for the whole school, such as the buddying programs or peer networks, this is manifested by the positive attitudes and participation of the general education classroom teachers. The anecdotal evidence by Hochman et al. (2015) is seemingly an example of this. In relation to CoF, Taylor's observation cited above emphasizes the teacher's style as one of the keys to success. Although an external agent is strongly recommended to allow children to speak honestly, the teacher is also requested to participate throughout the intervention process. However, Taylor's suggestions are only followed up in part of the reviewed CoF applications. In addition, references should be made to the involvement of external experts such as the researchers themselves. These professionals bring knowledge to the school by promoting the intervention in and of itself as well as training the facilitators (e.g., reported by Gardner et al., 2014). To be successful, intervention actions must derive from previous knowledge of two kinds. First, the corpus of scientific knowledge from research in the field. Second, knowledge already existing in the school, of problems, identified needs, strategies proved to be successful, and those unsuccessful, etc. This empiric knowledge comes from school staff, families and students, and should be the starting point for intervention in order to be effective in improving the school ethos. Hence, the setting up of a peer support program implies the necessary involvement of the school staff itself. When only the researchers implement the intervention, this seems to be less efficient.

Finally, families' support toward the peer support programs was hardly documented, although their commitment is markedly an important factor for successful education (Eccles and Harold, 1996; Bouffard and Stephen, 2007). Taken together, it is strongly recommended that research on the impact of peer support systems includes variables that represent these contributing factors of school and family support.

The reduced number of studies that were traced in the present review do not allow for firm conclusions, pointing at the need for more investigation on emotional peer support practices for SEND students. Still, the included documents covered a varied set of elaborated studies that allowed for several observations which might be useful for practitioners and researchers planning new initiatives for school ethos improvement through the valuable participation of peers. Improving relationships between students does not function without the involvement of the peers themselves.

## Data Availability Statement

The original contributions presented in the study are included in the article/supplementary material, further inquiries can be directed to the corresponding author/s.

## Author Contributions

KM, LG, and CB contributed to conception, design of the study, reviewed studies in relation to study's aims, and wrote sections of the manuscript. KM completed the systematic search for studies and wrote the first draft of the manuscript. All authors contributed to manuscript revision, read, and approved the submitted version.

## Conflict of Interest

The authors declare that the research was conducted in the absence of any commercial or financial relationships that could be construed as a potential conflict of interest.

## Publisher's Note

All claims expressed in this article are solely those of the authors and do not necessarily represent those of their affiliated organizations, or those of the publisher, the editors and the reviewers. Any product that may be evaluated in this article, or claim that may be made by its manufacturer, is not guaranteed or endorsed by the publisher.
